# ALPK1-Associated ROSAH Syndrome in a Polish Pedigree

**DOI:** 10.3390/genes17080870

**Published:** 2026-07-26

**Authors:** Agata Pietras-Baczewska, Adam Chmiel, Katarzyna Ognik, Robert Rejdak, Katarzyna Nowomiejska

**Affiliations:** 1 Department of General and Pediatric Ophthalmology, Medical University of Lublin, 20-079 Lublin, Poland; agatapie@gmail.com (A.P.-B.); robert.rejdak@umlub.edu.pl (R.R.); 2 Department of Ophthalmology, National Medical Institute of the Ministry of the Interior and Administration, 02-507 Warsaw, Poland; adam.t.chmiel@gmail.com; 3 Department of Biochemistry and Toxicology, Faculty of Animal Sciences and Bioeconomy, University of Life Sciences in Lublin, 20-950 Lublin, Poland; katarzyna.ognik@up.edu.pl

**Keywords:** ROSAH syndrome, retinal dystrophy, *ALPK1* gene

## Abstract

ROSAH syndrome is a rare autosomal dominant autoinflammatory disorder caused by gain-of-function mutations in the *ALPK1* gene, characterized by retinal dystrophy, optic nerve oedema, splenomegaly, anhidrosis, and headache. We present three related female patients (two sisters and their aunt) with a long-standing history of decreased visual acuity, recurring macular oedema, and progressive retinal dystrophy, as well as systemic involvement including anhidrosis, splenomegaly, headache, and arthritis. All our patients come from one family, with a total of 11 people suffering from ophthalmologic diseases with profound vision loss. Described three patients had confirmed pathogenic variant c.710C>T; p.Thr237Met in the *ALPK1* gene and extensive ophthalmic assessment. This case series highlights the importance of genetic testing and multimodal imaging in unexplained familial retinal dystrophies and underlines intrafamilial phenotypic variability. Moreover, it emphasizes ROSAH syndrome as an important differential diagnosis for inherited inflammatory vitreoretinopathies.

## 1. Introduction

ROSAH syndrome is a rare inherited autoinflammatory disease first described in 2012 as a set of symptoms including retinal dystrophy, optic nerve oedema, splenomegaly, anhidrosis, and headache [1]. The syndrome is caused by gain-of-function mutations in the *ALPK1* gene (alpha kinase 1) [2], which plays an important role in the elimination of pathogens and the activation of the adaptive immune system and contributes to constitutive activation of the NF-κB inflammatory pathway [3]. Alpha kinase 1 initiates an innate immune response by detecting bacterial pathogen-associated molecular pattern metabolites (PAMPs) and triggering a signaling cascade that leads to the activation of NF-κB and the secretion of inflammatory cytokines. The chronic inflammation results in a constellation of clinical features affecting multiple organ systems, with particularly severe ocular manifestations [4].

The most described *ALPK1* variant in patients with ROSAH syndrome is c.710C>T, p.Thr237Met, although c.761A>G, p.Tyr254Cys, c.830 C>T, and p.Ser277Phe [5] have also been described. So far, only pathogenic missense variants (primarily p.Thr237Met and p.Tyr254Cys) have been identified as causative for ROSAH syndrome [5,6].

The ocular phenotype of ROSAH syndrome is often the most prominent manifestation and is characterized by progressive bilateral retinal dystrophy with photoreceptor degeneration, optic nerve head edema, narrowed retinal vessels, and vitreous cellular infiltration [7]. These changes lead to progressive visual loss, often beginning in childhood or early adulthood. Systemic manifestations include splenomegaly, anhidrosis, recurrent headaches, and various cytopenias [1].

The inheritance pattern is autosomal dominant with variable penetrance, meaning affected family members may present with different severity of symptoms [2]. Recent advances in genetic testing have enabled more rapid diagnosis of this condition, which was previously often misdiagnosed as other forms of inherited retinal dystrophy or chronic uveitis [5]. Early diagnosis is crucial, as emerging biologic therapies show promise in controlling the inflammatory component of the disease [8]. Literature reports describe poor or incomplete control with corticosteroids and conventional immunosuppressants in most patients. The biological approach, IL-6 blockade, has been reported to produce striking systemic anti-inflammatory effects, and, in some cases, improvement in ocular inflammatory manifestations such as macular oedema, vasculitis, or optic disc swelling [1,2]. The full clinical spectrum and long-term course of ROSAH syndrome remain incompletely defined. To date, fewer than 80 cases of ROSAH syndrome have been reported in the literature [9].

In this article, we present three related female patients with genetically confirmed ROSAH syndrome, who were initially diagnosed with inflammatory vitreoretinopathy.

## 2. Methods

### 2.1. Ophthalmic Examination

Two of the described patients were selected from 204 participants of the Lublin Digital Union—Use of Digital Solutions and Artificial Intelligence in Medicine—Research Project founded by the Polish Ministry of Education and Science, grant no. MEiN/2023/DPI/2194. The study adhered to the tenets of the Declaration of Helsinki and was formally approved by the local Institutional Ethics Committee of the Medical University of Lublin (nr KE-0254/260/12/2022). Written informed consent was obtained from the parents prior to data collection. Inclusion criteria consisted of the identification of a pathogenic *ALPK1* mutation along with systemic and ophthalmologic clinical features supporting the diagnosis of ROSAH syndrome.

Medical charts were meticulously reviewed to ensure maximum data integrity. Longitudinal clinical data were extracted and included demographics and a complete ophthalmological evaluation including at least best-corrected visual acuity using Snellen charts (BCVA, in decimal), and findings from both anterior segment and dilated fundus examinations.

Patients underwent follow-up visits, during which comprehensive multimodal imaging was systematically performed to monitor structural changes. Morphological evaluation included spectral-domain optical coherence tomography (SD-OCT), initially acquired with a Zeiss Cirrus 6000 OCT (Carl Zeiss Meditec Inc., Dublin, CA, USA). Wide-field fundus autofluorescence (FAF) and color fundus photography were performed using an Optos fundus camera (Optos plc, Dunfermline, Scotland, UK). Visual field examination was performed using a Humphrey Field Analyser II (Carl Zeiss Meditec Inc., Dublin, CA, USA) and revealed concentric constriction of the visual field to less than 20 degrees. Electrophysiology examination of the included patients was performed in another ophthalmological department and showed substantial reductions in both dark-adapted (scotopic) and light-adapted (photopic) a- and b-wave amplitudes, indicating widespread photoreceptor dysfunction.

### 2.2. Pedigree of the Family

**Figure 1:**
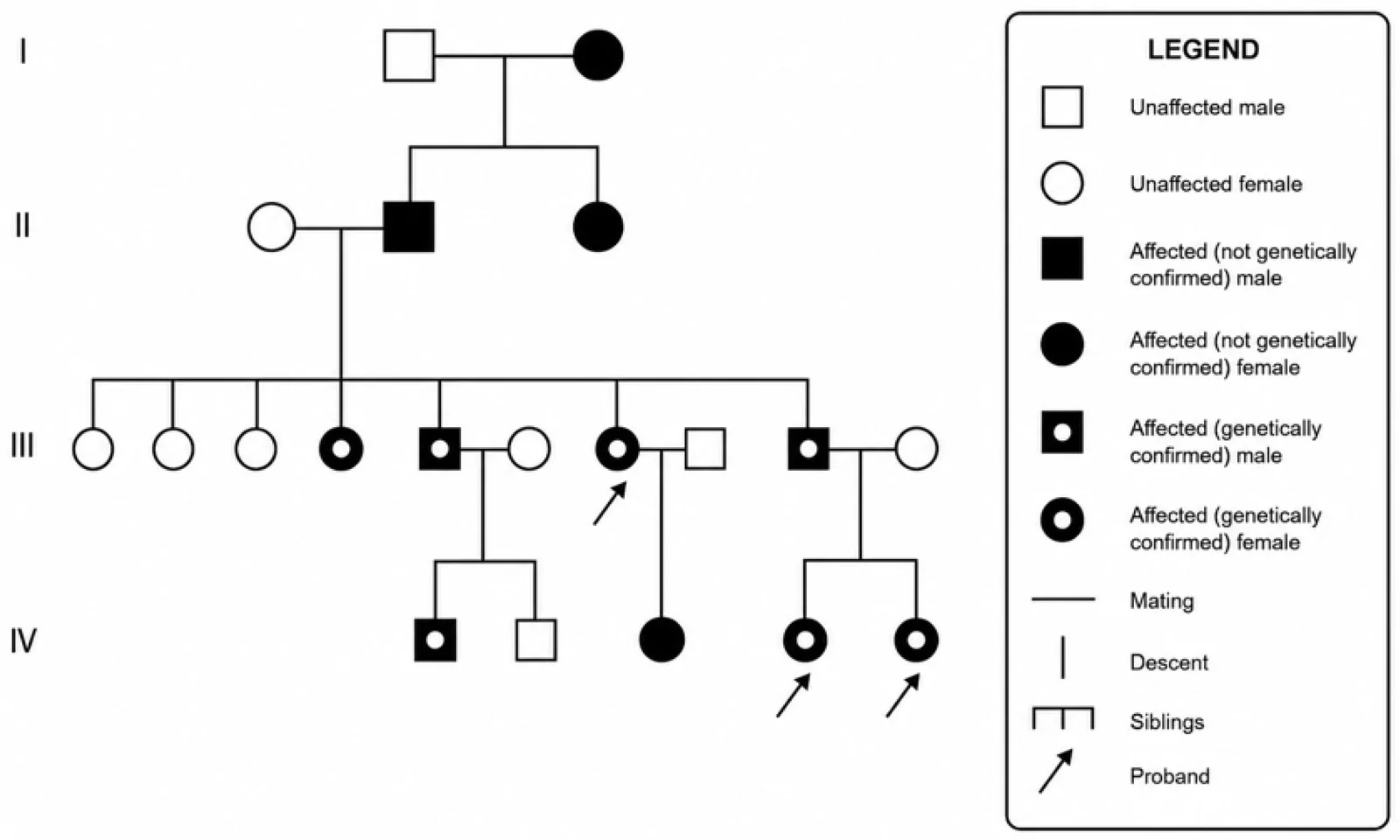
Family pedigree chart.

All of our patients are from one family, which includes a total of 11 people suffering from ophthalmologic diseases with profound vision loss. All living family members—seven people—with ophthalmological syndromes had ROSAH syndrome confirmed via molecular testing conducted at various medical centers worldwide. As stated by our patients, there were at least 3 or 4 people blind at a young age in older generations, and a teenaged girl with ROSAH syndrome symptoms, who passed away tragically before genetic testing (Figure 1).

### 2.3. Genotyping Analysis

Genetic testing of patients 1 and 2 was performed at CeGaT genetic laboratory in Tübingen, Germany. Genomic DNA was extracted from the peripheral blood of each participant using a commercial QIA Symphony DSP DNA Mini Kit (Qiagen N.V., Venlo, The Netherlands) according to the manufacturer’s protocol and subsequently quantified on a Gemini XPS Dual-Scanning Microplate Spectrofluorometer (Molecular Devices Corporation, Sunnyvale, CA, USA). The extracted DNA was used for whole-exome sequencing (WES) on the Illumina NovaSeq6000 platform (Illumina, Inc., San Diego, CA, USA), following target enrichment via solution-based hybridization using a custom WES panel (CeGaTExomeXtra^®^ v.5), developed by Twist Bioscience (South San Francisco, CA, USA). Additionally, known non-coding pathogenic variants (UTR, intronic, and intergenic) were enriched and assessed according to ClinVar, HGMD, and CeGaT’s internal database. Both blood and extract DNA were disposed of immediately after completion of the genetic analysis. Sequencing reads were processed using bcl2fastq2, aligned to the reference genome hg19 (Skewer, BWA), and analyzed for SNVs, small indels, and copy number variations (CNVs). Internal panels for RP, consistent with the patients’ phenotypes, were analyzed in silico. Variant interpretation was based on the clinical information available at the time of analysis, and variants were classified according to ACMG/ACGS 2020 guidelines (version 4.01).

### 2.4. Cases Presentation

#### 2.4.1. Case 1

The first patient in this case series is a 24-year-old female with a positive family history of vitreoretinopathy presented with long-standing history of ocular inflammation and visual impairment. Ocular abnormalities were first noted at the age of 6, when fundus examination revealed blurred optic disc margins and macular oedema, accompanied by inflammatory changes along retinal vessels. Throughout childhood and adolescence, the patient experienced recurrent episodes of macular oedema and panuveitis-like symptoms. By 2016, she was initially diagnosed with familial neovascular inflammatory vitreoretinopathy, with suspected CAPN5-associated disease, based on clinical presentation and autosomal dominant inheritance pattern. No genetic testing was performed at the time.

In 2021, the best-corrected visual acuity (BCVA) was 0.5 in both eyes. Fundus examination revealed pale and slightly narrowed optic discs, vitreous opacities, and an abnormal macular reflex.

In July 2022, cranial computed tomography demonstrated diffuse hypodense changes in the left frontal lobe, possibly of vascular origin, raising concerns for systemic manifestations of an underlying autoinflammatory disorder. Later in 2022, the patient presented to Emergency Department with acute bilateral vision loss and headache. Examination revealed elevated intraocular pressure (34 mmHg bilaterally) and shallow anterior chambers. BCVA was limited to questionable light perception in the right eye and 0.05 in the left eye. Acute angle-closure glaucoma was diagnosed, and systemic acetazolamide was administered, resulting in symptomatic improvement. Laser peripheral iridotomy was subsequently performed in both eyes, followed by topical antiglaucoma therapy. In follow-up examinations, intraocular pressure normalized and visual acuity improved (up to 0.5 in both eyes). Fundus examination confirmed ongoing vitreoretinal pathology, including persistent vitreous opacities, RPE abnormalities, peripheral retinal degeneration, and peripapillary nerve fiber layer oedema.

Given the atypical course and the presence of extraocular manifestations, a syndromic condition was suspected. The patient reported a history of recurrent headaches, arthritis, dental abnormalities, altered salivary consistency (thick saliva), and anhidrosis. Considering this constellation of findings, genetic testing was performed in 2024 in Cegat laboratory (Tübinegen, Germany). This identified a pathogenic *ALPK1* variant (c.710C>T; p.Thr237Met), establishing the diagnosis of ROSAH syndrome. In 2025, she had initiated biologic therapy with tocilizumab following international consultation.

**Figure 2:**
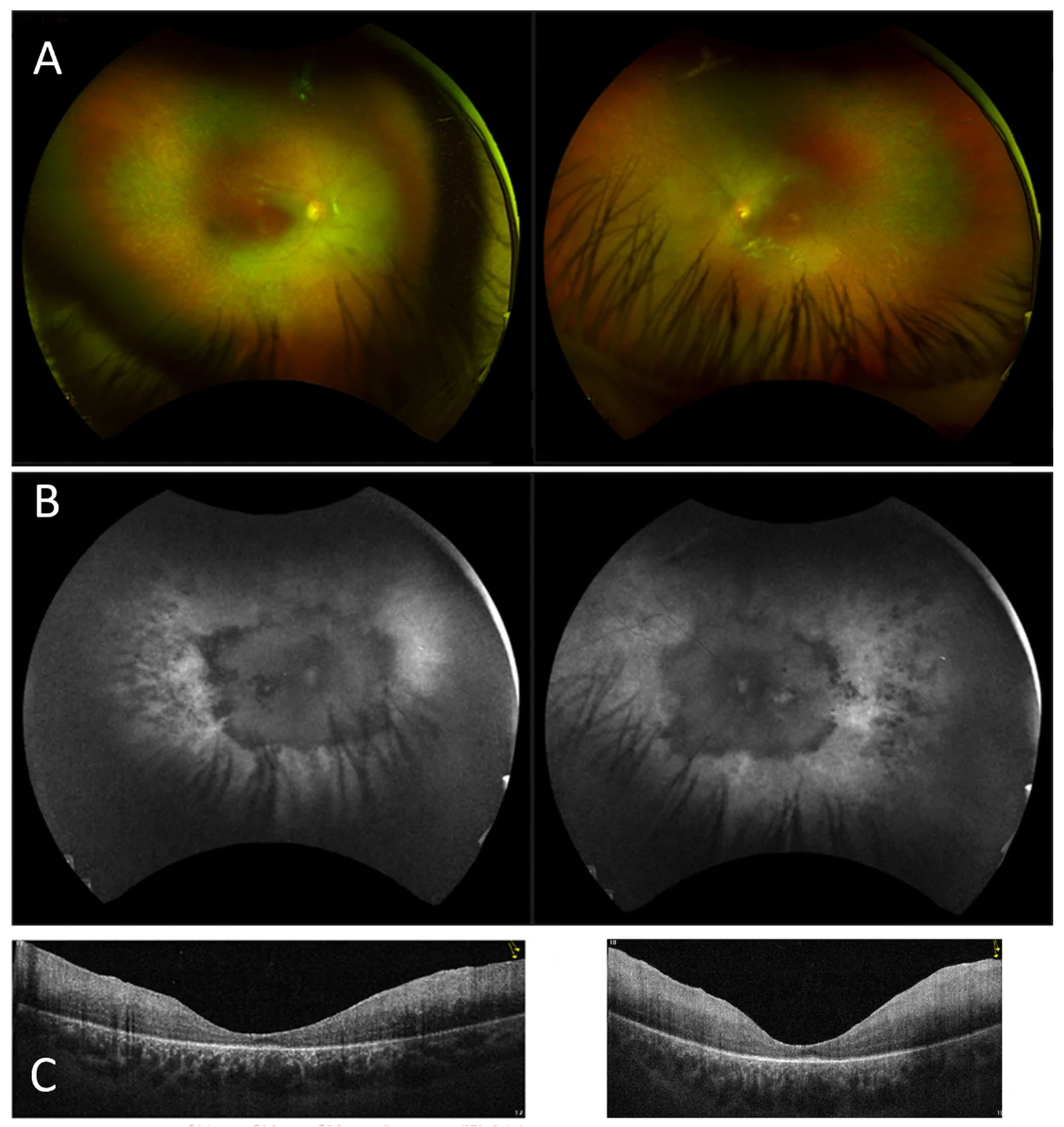
Patient 1, Ultra-widefield fundus images of both eyes: (**A**) color fundus photography, (**B**) fundus autofluorescence, (**C**) OCT macula scan.

By May 2026, the patient remained under follow-up with relatively stable visual acuity (0.4 in the right eye and 0.5 in the left eye) and controlled intraocular pressure. Multimodal imaging was performed; ultra-widefield imaging revealed diffuse retinal degeneration with attenuation of retinal vessels, areas of RPE disturbance, peripapillary nerve fiber layer oedema, and hazy media due to vitreous opacities (Figure 2A).

FAF images revealed a bilateral ring of hyperautofluorescence surrounding an area of isoautofluorescence with further central hyperautofluorescence (Figure 2B).

Optical coherence tomography (OCT) demonstrated foveal profile abnormality, outer retinal atrophy with disruption of the ellipsoid zone and thinning of photoreceptor layers, and diffuse hyper-reflectivity of the inner retina in both eyes. (Figure 2C).

#### 2.4.2. Case 2

The second patient was a 27-year-old female who exhibited a more severe and rapidly progressive disease course than her younger sister.

She was first diagnosed in 2006, at 6 years of age, with decreased visual acuity (0.3 in the right eye and 0.5 in the left eye with hyperopic correction). Fundus examination revealed an optic disc oedema, macular elevation, and inflammatory changes along retinal vessels.

Throughout childhood and adolescence, the patient had recurrent macular oedema with accompanying intraocular inflammation affecting multiple structures, resembling panuveitis. These were treated with systemic corticosteroid therapy and periocular triamcinolone injections, which provided only temporary improvement. Over time, progressive visual deterioration occurred, with BCVA declining to 0.2 in both eyes in 2010. Following a genetic consultation in 2016, she was diagnosed with familial neovascular inflammatory vitreoretinopathy, similarly to her younger sister and father.

Despite treatment, the disease progressed. By 2020, BCVA had declined to 0.05 in both eyes. The patient underwent cataract surgery with PCIOL implantation in the left eye. Fundus examination was limited due to vitreous opacities, but persistent retinal abnormalities were evident. In 2023, further deterioration occurred and the patient required cataract surgery in the right eye. Despite successful cataract surgeries in both eyes, VA declined to light perception in the right eye and counting fingers at 2m in the left eye.

Similarly to her sister, the second patient exhibited similar extraocular manifestations suggestive of a systemic disorder. These included headaches, arthritis, dental abnormalities, altered salivary consistency (thick saliva), and hypohidrosis. Notably, splenomegaly was also documented, representing a key systemic feature consistent with the ROSAH phenotype. Genetic testing ultimately confirmed the same *ALPK1* pathogenic variant (c.710C>T; p.Thr237Met), establishing the diagnosis of ROSAH syndrome (Cegat, Tübingen, Germany).

**Figure 3:**
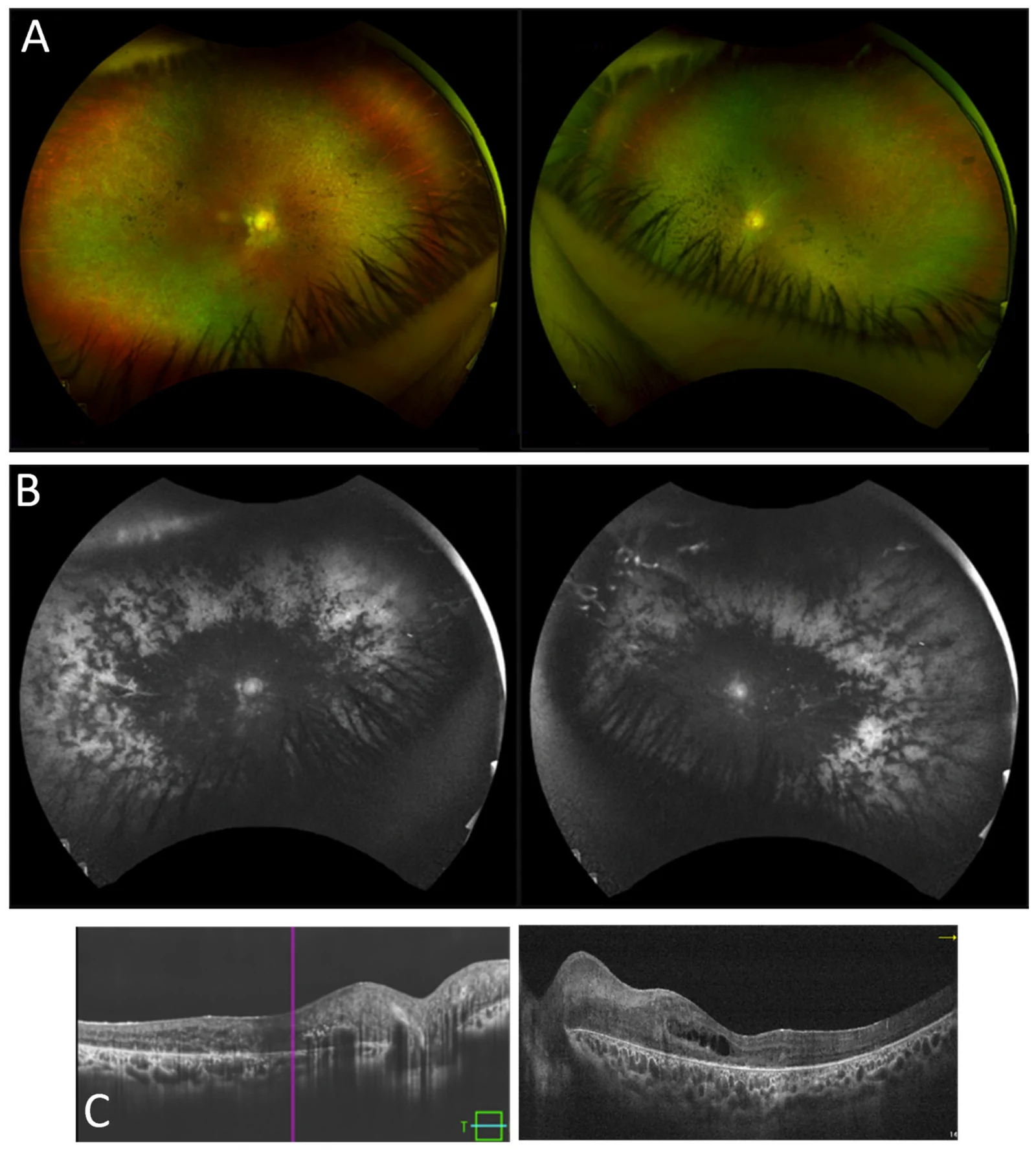
Patient 2, Ultra-widefield fundus images of both eyes: (**A**) color fundus photography, (**B**) fundus autofluorescence, (**C**) OCT scan of the macula.

Similarly to her sister, since 2025, she has been undergoing biologic therapy with tocilizumab. By May 2026, BCVA remained profoundly impaired (light perception in both eyes), indicating advanced retinal degeneration with photoreceptor loss. Ultra-widefield fundus imaging demonstrated advanced bilateral retinal degeneration with extensive RPE atrophy, severe attenuation of retinal vasculature, dense vitreous opacities with organised posterior vitreous detachment, and prominent pigmentary changes (Figure 3A).

Fundus autofluorescence imaging revealed bilateral, symmetric extensive hypoautofluorescent areas involving the posterior pole and mid-periphery, consistent with widespread RPE and outer retinal degeneration (Figure 3B).

OCT of the macula in both eyes revealed near-complete loss of the ellipsoid zone, profound outer nuclear layer thinning, and an irregular foveal contour. In the left eye, intraretinal cystic spaces were noted (Figure 3C).

#### 2.4.3. Case 3

The third patient, a 52-year-old female, is the aunt of the two previous patients (their father’s sister). She was first diagnosed with bilateral atypical optic neuritis as a teenager in the early 1990s, with decreased visual acuity 0,1 OD, 0,16 OS. Fundus examination revealed an optic disc oedema, which was followed by intravenous steroids. Throughout adolescence, the patient had recurrent intraocular inflammation affecting multiple structures, resembling panuveitis. These were treated with systemic corticosteroids, which provided only temporary improvement. Over time, progressive visual deterioration occurred with BCVA declining to 0.2 in both eyes in 2010. As the result of recurrent uveitis, she developed anterior synechiae with mature cataract and advanced fundus dystrophy. The patient discontinued treatment in our department for many years.

Genetic testing from 2024 performed in the National Human Genome Research Institute in Bethesda (MD, USA) ultimately detected an *ALPK1* c.710C>T; p.Thr237Met pathogenic variant, confirming the ROSAH syndrome diagnosis. Like the sisters described above, since April 2025 she has been undergoing biologic therapy with tocilizumab, coordinated by rheumatology specialists.

The patient re-presented at the emergency room in November 2025 with bacterial keratitis in the right eye. Visual acuity was light perception in both eyes. The ophthalmological examination revealed corneal opacity, keratoconus, hypopyon, posterior synechiae, and mature cataract with no insight to vitreous cavity or retina. The left eye showed no inflammation, a clear anterior chamber, and aphakia. The mature cataract had dislocated to the vitreous cavity, still attached by remaining zonules.

**Figure 4:**
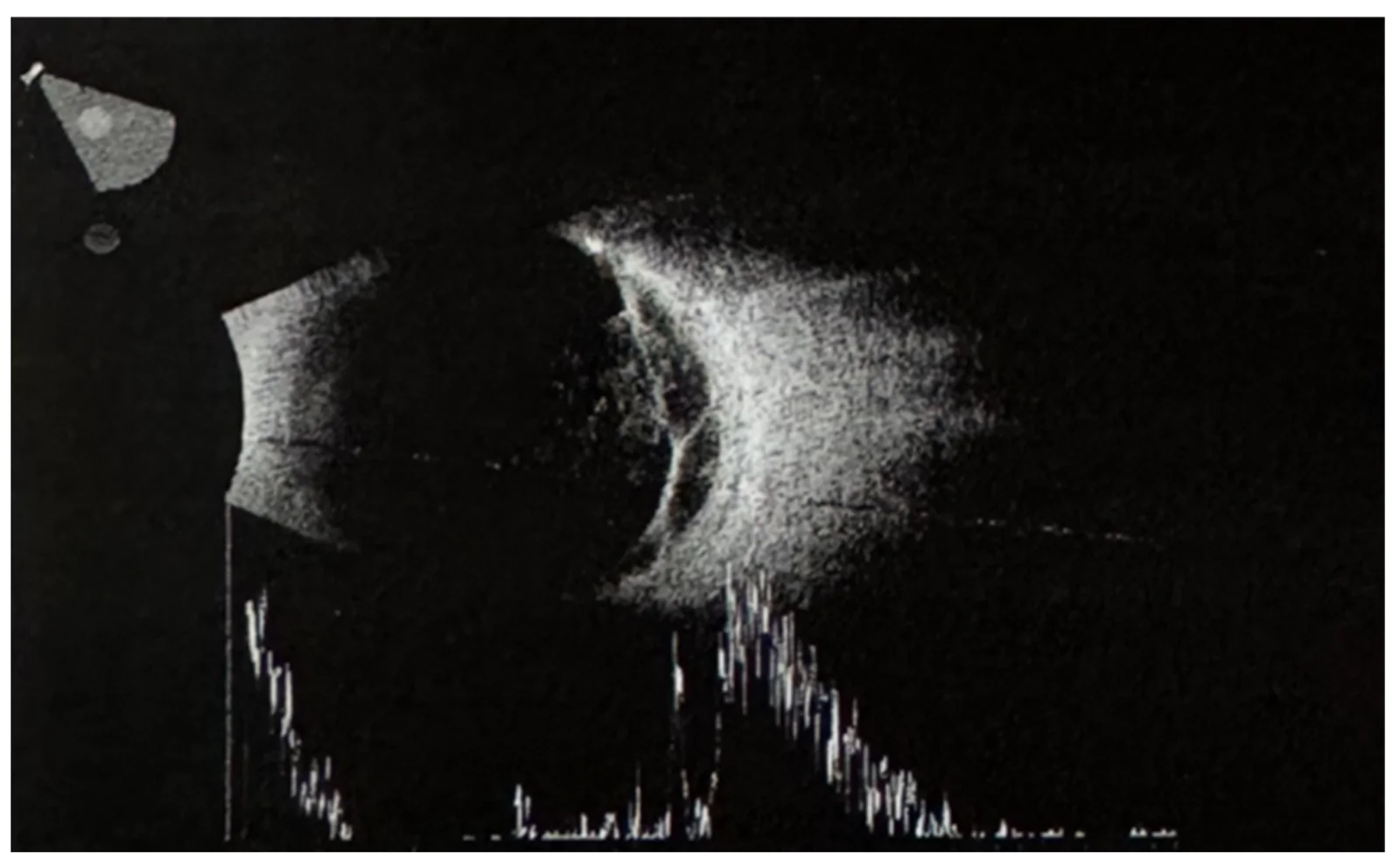
Ultrasound scan of the right eye of patient 3.

At the most recent follow-up visit in May 2026, visual acuity was light perception in both eyes. The ophthalmological examination was the same as during previous visits. Additional ultrasound scan showed opacities in the vitreous chamber and post-inflammatory vitreoretinopathy with tractional retinal detachment (Figure 4).

**Figure 5:**
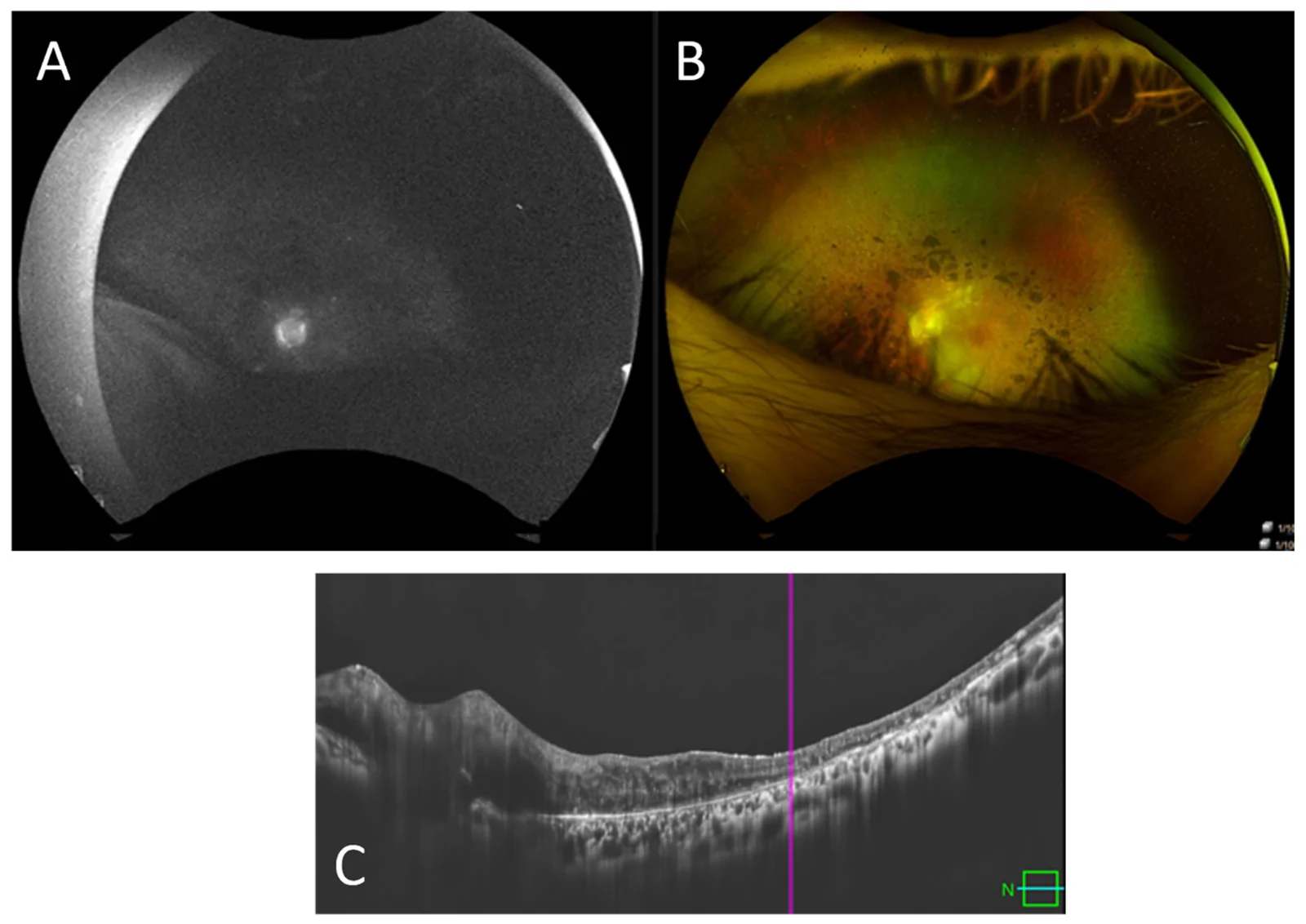
Patient 3. Ultra widefield fundus autofluorescence (**A**), color photography (**B**) and OCT scan of the macula (**C**) of the left eye.

Ultra-widefield fundus imaging demonstrated advanced retinal degeneration with RPE atrophy, severe attenuation of retinal vasculature, vitreous opacities, blurred margins of the optic disc, and prominent pigmentary changes (Figure 5A). FAF imaging revealed extensive hypoautofluorescent areas involving the posterior pole and mid-periphery, consistent with widespread RPE and outer retinal degeneration (Figure 5B). Due to the advanced lens opacification, it was not possible to obtain fundus photography of the fellow eye.

OCT of the macula demonstrated foveal profile abnormality, outer retinal atrophy with disruption of the ellipsoid zone, and photoreceptor layers thinning in the left eye (Figure 5C). It was impossible to obtain a scan of the right eye due to the mature cataract.

**Figure 6:**
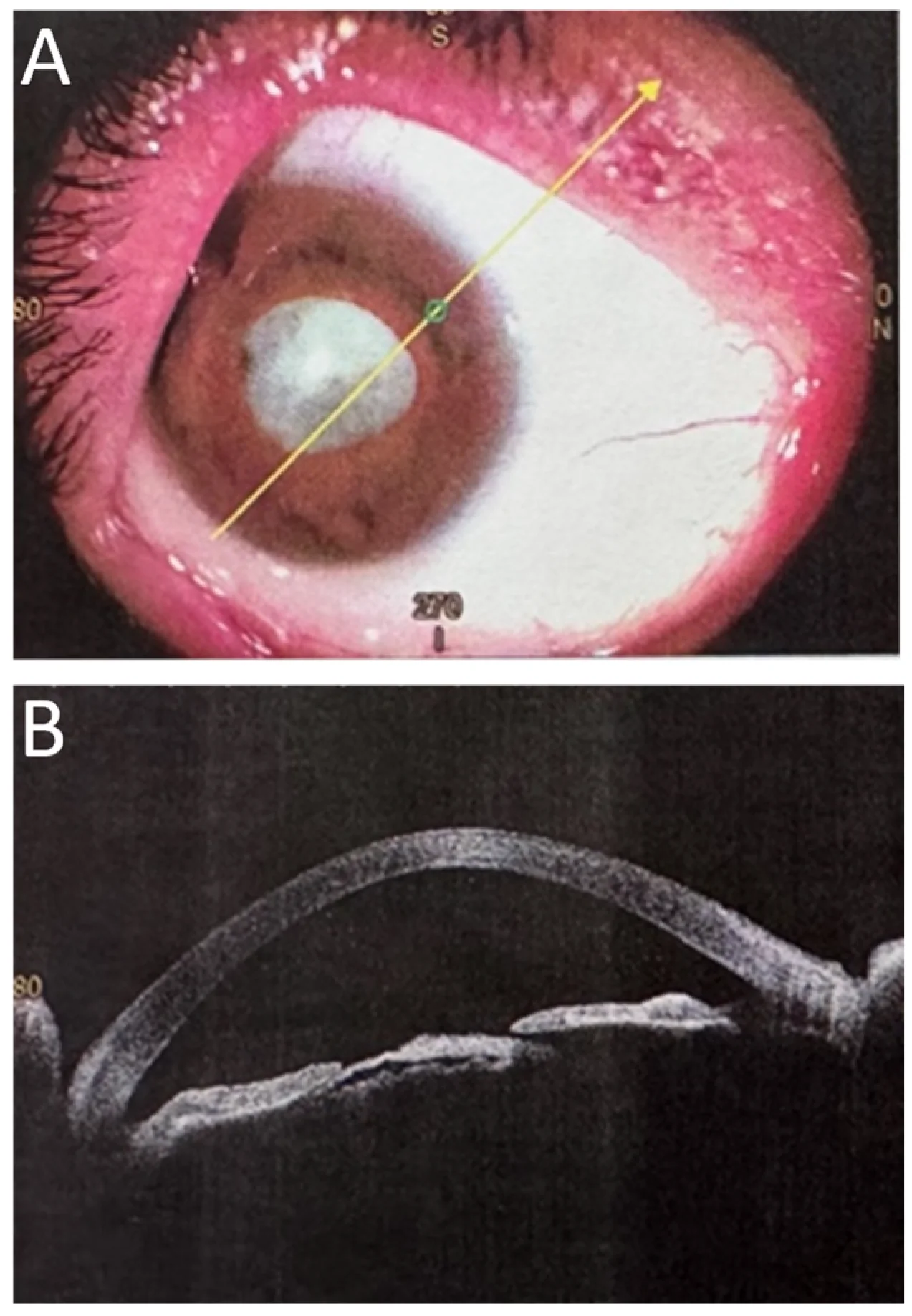
Anterior segment photography (**A**) and AS-OCT (**B**) of the right eye of the third patient.

Anterior segment photography indicated neither irritation nor anterior segment inflammation on the examination day. However, there was a mature cataract with posterior synechiae. The AS-OCT scan showed a clear anterior chamber and iris-lenticular adhesions (Figure 6).

## 3. Discussion

This case series highlights the diagnostic and clinical challenges associated with ROSAH syndrome. ROSAH syndrome results from gain-of-function in the *ALPK1* gene, which encodes alpha-protein kinase 1, a member of the atypical kinase family involved in innate immunity. *ALPK1* localizes to the photoreceptor connecting the cilium, retinal pigment epithelium, and centrosomes. It has been demonstrated that pathogenic *ALPK1* variants lead to constitutive activation of the NF-κB inflammatory pathway, resulting in chronic, uncontrolled inflammation [3]. This molecular mechanism distinguishes ROSAH syndrome from purely degenerative retinal dystrophies and provides a rationale for targeted anti-inflammatory therapy. Snelling et al. recently identified and functionally characterized a novel *ALPK1* variant, further expanding our understanding of the genetic heterogeneity in ROSAH syndrome [10]. Sun et al. described how different *ALPK1* mutations may correlate with varying ocular phenotypes, suggesting that genotype–phenotype correlations may help predict disease severity and progression [11].

Two sisters in our series were initially diagnosed with familial neovascular inflammatory vitreoretinopathy, with suspected CAPN5-related disease. This reflects a well-recognized diagnostic pitfall, as ROSAH syndrome can mimic other inherited retinal disorders characterized by inflammation and autosomal dominant inheritance [4]. The clinical features in both our patients were early-onset, recurrent intraocular inflammation affecting multiple ocular structures, including the retina, choroid, and optic nerve. This group of symptoms closely mimicked CAPN5-associated vitreoretinopathy, leading to years of misdiagnosis. For many years, the oldest patient in our group, the aunt, was not ophthalmologically controlled, because she had already lost her vision, and there is still no targeted treatment. While none of our patients experienced vitreous hemorrhage, Hong et al., in their multimodal imaging study, recently reported this as a potential complication of the syndrome, emphasizing the need for regular follow-ups of patients to monitor disease progression [12]. Our patients showed distinct ocular involvement, which was the first manifestation of the disease.

A striking finding in this series is the marked intrafamilial variability in disease severity. Despite a shared genetic background, the second patient experienced a significantly more aggressive course, with rapid progression to severe visual impairment, structural retinal damage, and systemic involvement with splenomegaly, while first patient retained relatively preserved visual function and demonstrated less pronounced systemic involvement. Structural OCT findings correlated with functional status, with more preserved retinal architecture in the first patient and advanced photoreceptor degeneration in the second one. Variable expressivity and incomplete penetrance of the mutation may explain this differences in disease severity; however, further investigation is needed. The oldest patient had the lowest visual acuity. Possibly, this could be due to different disease progression with regard to retinal anterior chamber abnormalities, advanced cataract at a young age, and uveitis. Meanwhile, her right eye was probably left uncontrolled and untreated with an unoperated mature cataract and proliferative vitreoretinopathy leading to tractional retinal detachment.

Genetic testing played a decisive role in establishing the diagnosis in all three cases. As emphasized in the recent literature [13], early identification of *ALPK1* mutations is critical, not only for accurate diagnosis but also for potential therapeutic implications. Traditional immunosuppression with corticosteroids provided only temporary benefit and failed to prevent progressive retinal degeneration in the second patient, consistent with previous reports showing limited efficacy of conventional anti-inflammatory therapy. Emerging evidence suggests that targeted biologic therapies, particularly interleukin-6 inhibitors, may help control inflammation, stabilize retina degeneration and potentially modify disease progression. The IL-6 inhibitor, tocilizumab, is effective at reducing macular oedema associated with uveitis [14] and may also be beneficial for treating macular edema in the setting of retinal degeneration [15]. In addition to regulating T- and B-cell development and activation, NF-κB also controls the expression of pro-inflammatory cytokines in adaptive immunity. When immune cells are infected or damaged, NF-κB is activated and induces the synthesis of various pro-inflammatory cytokines, such as tumor necrosis factor-alpha (TNF-α), interleukin-1 beta (IL-1β), and interleukin-6 (IL-6). These cytokines can trigger inflammatory reactions and attract other immune cells to eliminate pathogens or repair damaged tissues [15].

In our case, patients were treated with tocilizumab, an interleukin-6 (IL-6) receptor inhibitor, following genetic confirmation of ROSAH syndrome. The IL-6 treatment was initiated by the coordinating rheumatologist in a different department, lasting approximately one year for all patients. The patients report that symptoms of other systems affected by ROSAH have improved since the tocilizumab therapy. However, the ophthalmological response appears to have depended strongly on the stage of disease at treatment initiation. The first patient, with a relatively preserved retinal structure, achieved stabilization of visual acuity and disease progression following initiation of tocilizumab. In contrast, the two consecutive patients, in whom treatment was initiated at an advanced stage with significant photoreceptor loss and extensive retinal degeneration, showed limited functional improvement despite therapy. This observation supports the hypothesis that early initiation of targeted therapy may be critical for preventing irreversible retinal damage, emphasizing the importance of prompt diagnosis and early targeted intervention. The optimal timing, dosing, and duration of biologic therapy for ROSAH syndrome remain unclear. Early intervention may be critical, as advanced photoreceptor loss as seen in second patient is unlikely to be reversible with currently available treatments. Prospective studies are needed to determine whether biologic therapy can slow or prevent photoreceptor degeneration when initiated early in the disease course.

This report is limited by the small number of patients and a relatively short follow-up after initiation of biologic therapy. Another shortcoming is that functional testing including electroretinography was not available throughout the follow-up; it was performed at baseline in another ophthalmological department.

Tornero-Romero and colleagues emphasized the importance of establishing screening protocols for ROSAH syndrome in patients presenting with hyperinflammation and unexplained visual loss [16]. Based on our experience and the growing literature, we recommend that ROSAH syndrome should be considered as a differential diagnosis in patients with early-onset, familial retinal dystrophy accompanied by recurrent intraocular inflammation and optic nerve involvement, particularly when inheritance follows an autosomal dominant pattern.

Early recognition of ROSAH syndrome is increasingly important in light of emerging therapies that may alter the disease course and improve patient outcomes. Timely molecular diagnosis and initiation of targeted biologic therapy may help preserve retinal structure and visual function before irreversible photoreceptor degeneration occurs. As our understanding of ROSAH syndrome grows and treatment possibilities evolve, coordinated multidisciplinary care involving ophthalmology, rheumatology, and genetics will be crucial for optimizing outcomes in affected patients.

## Data Availability

The original contributions presented in this study are included in the article. Further inquiries can be directed to the corresponding authors.
